# Increasing Onshore Oil Production: An Unexpected Explosion in Trauma Patients

**Published:** 2018-05-18

**Authors:** Dakota M. Urban, Jeanette G. Ward, Stephen D. Helmer, Alan D. Cook, James M. Haan

**Affiliations:** 1 University of Kansas School of Medicine-Wichita, Department of Surgery; 2 Chandler Regional Medical Center, Chandler, AZ; 3 Via Christi Hospital Saint Francis, Wichita, KS

**Keywords:** oil and gas industry, oil and gas fields, trauma, wounds and injuries, safety

## Abstract

**Introduction:**

Few data currently exist which are focused on type and severity of onshore oil extraction-related injuries. The purpose of this study was to evaluate injury patterns among onshore oil field operations.

**Methods:**

A retrospective review was conducted of all trauma patients aged 18 and older with an onshore oil field-related injury admitted to an American College of Surgeons-verified level 1 trauma center between January 1, 2003 and June 30, 2012. Data collected included demographics, injury severity and details, hospital outcomes, and disposition.

**Results:**

A total of 66 patients met inclusion criteria. All patients were male, of which the majority were Caucasian (81.8%, n = 54) with an average age of 36.5 ± 11.8 years, injury severity score of 9.4 ± 8.9, and Glasgow Coma Scale score of 13.8 ± 3.4. Extremity injuries were the most common (43.9%, n = 29), and most were the result of being struck by an object (40.9%, n = 27). Approximately one-third of patients (34.8%, n = 23) were admitted to the intensive care unit. Nine patients (13.6%) required mechanical ventilation while 27 (40.9%) underwent operative treatment. The average hospital length of stay was 5.8 ± 16.6 days, and most patients (78.8%, n = 52) were discharged home. Four patients suffered permanent disabilities, and there were two deaths.

**Conclusion:**

Increased domestic onshore oil production inevitably will result in higher numbers of oil field-related traumas. By focusing on employees who are at the greatest risk for injuries and by targeting the main causes of injuries, training programs can lead to a decrease in injury incidence.

## Introduction

In the United States (U.S.) between 2003 – 2013, the oil and gas extraction industry experienced a 71% increase in the number of active oil rigs.^1^ Onshore based operations involving horizontal drilling and fracturing experienced the greatest growth, seeing an increase in employment rates between 40% to 92%.^1^–^4^ One place in particular that saw an increase in the number of onshore rigs due to the success rate of horizontal drilling and hydraulic fracturing operations was Kansas.^5^ Although this increase was not as high as rates seen in Texas and Oklahoma, Kansas saw the addition of 1,000 active wells during this time.

In 2011, 1,400 workers directly involved in operating and developing oil and gas field properties and 8,500 workers involved in support activities were injured on the job.^6^ Most of these injuries, regardless of whether they were employed at an on- or off-shore facility, were related to highway motor vehicle crashes or extreme impact/crush.^6^ However, explosions and flash fires on onshore rigs have become common due to the increased use of fracturing.^4^ The median days-away-from-work for those injured while working at or near an oil rig has been reported as three times longer (24 days) compared to all other industries (8 days).^6^

The occupational fatality rate for this industry is four to seven times higher than among U.S. workers in general.^1^–^3^,^7^,^8^ The majority of oil and gas extraction-related fatalities are due to transportation incidents and contact with objects or equipment.^1^,^3^,^7^,^8^ Factors that may increase the rate of injuries and the frequency of fatalities include working on aging rigs or, for smaller companies, length of time on the job, being subcontracted, or participating in rig maintenance, repairs, or drilling operations.^2^,^3^,^8^ Human error, equipment failure, and weak operating systems also were contributing factors.^9^,^10^

The majority of literature on the oil and gas extraction industry addresses the rate of offshore occupational related-injuries.^9^–^17^,^19^ A closer examination of injury patterns and outcomes among onshore drilling workers could prove beneficial for triage and treatment of the patient in the field and hospital settings, as well as illustrate the need for safety procedures to prevent injury in this industry. The purpose of this study was to evaluate injury patterns in onshore oil field operations.

## Methods

A retrospective review of all adult patients admitted with injuries sustained during the operation or maintenance of onshore oil field machinery between January 1, 2003 and June 30, 2012 was conducted at a single American College of Surgeons-verified level 1 trauma center. Data were retrieved from the trauma registry, as well as from each patient’s medical records. Patient data included age, sex, race, injury severity score (ISS), abbreviated injury severity score (AIS), Glasgow Coma Scale (GCS) score, and injury details. Hospitalization data included intensive care unit (ICU) admission and length of stay, mechanical ventilation requirements, and need for operative management. Outcomes data included hospital length of stay, discharge disposition (home, rehabilitation, skilled nursing facility), and mortality.

Descriptive analyses were presented as frequencies with percentages for categorical variables and means with standard deviations for continuous variables. All statistical analyses were conducted using SPSS release 19.0 (IBM Corp., Armonk, New York). This study was approved for implementation by the Institutional Review Board of Via Christi Hospitals Wichita, Inc. and the University of Kansas School of Medicine-Wichita’s Human Subjects Committee.

## Results

A total of 66 patients met the inclusion criteria for the study. All patients were male, and the majority were Caucasian (81.8%, n = 54) with an average age of 36.5 ± 11.8 years, ISS of 9.4 ± 8.9, and GCS of 13.8 ± 3.4 (Table 1). Based on AIS, the most severely injured body regions were the abdomen (2.7 ± 0.8) and the extremities (2.7 ± 0.7). All injuries were the result of blunt force trauma, and most were the result of being struck by an object (40.9%, n = 27). Falls (19.7%, n = 13) accounted for the second most common cause of injury, followed by caught in machine (12.1%, n = 8), and explosions (10.6%, n = 7).

Most injuries were to the extremities with lower extremity injuries being the most frequent (25.8%, n = 17; Table 2). Injuries to the head and face also were common, with most involving a facial fracture (22.7%, n = 15) or loss of consciousness (16.7%, n = 11). Among patients who sustained a vertebral spinal fracture, lumbar fractures (12.1%, n = 8) were the most common. Injuries to the thoracic and abdominal regions were not as common.

Slightly over one-third (34.8%, n = 23) of patients were admitted to the ICU with an average length of stay of 1.7 ± 2.5 days (Table 3). Mechanical ventilation was required for 13.6% (n = 9) of patients and 40.9% (n = 27) required surgery. The majority of surgical interventions involved debridement and open reduction of extremity fractures. In addition, four patients required completion of an amputation and one patient required multiple orthopedic and abdominal surgeries. The average hospital length of stay was 5.8 ± 16.6 days, and most patients (78.8%, n = 52) were discharged home. Four patients suffered a permanent disability, and two patients (3.0%) died due to explosion-related injuries.

## Discussion

With a marked increase in the number of active onshore oil rigs in the United States, there is a correlated increase in injury and fatality rates among oil and gas extraction workers.^1^,^8^ Although there is previous research for offshore oil rigs, there is no study that specifically focuses on onshore oil rig injury characteristics based on hospital data.^1^–^3^ In the current study, extremity fractures and head/facial injuries were the most common. In addition, the majority of injuries were due to the patient being struck by an object or as the result of a fall. The number of fatalities in the current study was low, and both were explosion related.

Our results supported several offshore drilling injury studies.^12^,^13^,^16^ For example, a study conducted among Venezuelan drillers indicated that most injuries were to the upper (48%) and lower (24%) extremities with the majority resulting from the worker being struck by an object (37%).^12^ Our study demonstrated lower rates of upper and lower extremity injuries, 25.8% and 18.2%, respectively; however, the type and cause of these injuries were similar, as was the fact that they were the most common. Another study of Iranian gas refinery workers demonstrated most injuries were caused by being struck by an object (48%).^13^ We reported a 40.9% rate of injury associated with being struck. In addition, Mehrdad^13^ and Thibodaux^16^ reported most injuries caused by an offshore drilling accident were to the extremities.

Fatality statistics from the Bureau of Labor Statistic (BLS) Census of Fatal Occupational Injuries (CFOI) were used for comparisons regarding patient fatality rates.^1^,^3^,^6^,^8^ Of note, it has been well documented that CFOI injuries are under-reported in this database.^17^,^18^ The BLS studies demonstrated that most fatal injuries were caused by transportation-related accidents (40%), followed by contact with objects and equipment (26%), fires and explosions (14%), and finally falls, slips, and trips (8%).^1^,^3^,^6^,^8^ In the current study, there were no transportation-related fatalities; the two reported deaths were explosion-related.

Possible fall prevention measures for our study population might include the use of a full body harness, impact protective clothing, or the use of personal fall arrest system (PFAS).^4^,^19^,^20^ To protect workers from dangerous machinery and prevent accidental contact with objects, the use of suitable covers or casings, and barrier rails or screens are needed.^20^–^21^ However, it has been documented that many onshore oil rigs routinely are unassembled and moved quickly resulting in design modifications that may involve removing handrails.^21^ Prevention of injuries from being struck by an object may include strongly enforcing Occupational Standard Health Administration (OSHA) personal protective equipment regulations and implementing penalties for workers caught not following these regulations.

Recommendations for future research include amalgamating hospital data with occupational reports to produce an accurate picture of which types of workers sustain the most severe injuries or are at the highest risk for death. For instance, Blakeley et al. 2 reported that improved engineering controls and safety programs would benefit floor men at a higher rate than other job types due to the fact they experience three times the rate of injuries compared to other positions. In addition, due to the small sample size of the current study, expanding beyond a single institution by including multiple hospitals would be beneficial for establishing injury patterns for onshore oil rigs.

This study had several limitations. First, the findings are limited by all known biases associated with retrospective studies. These include a lack of granularity that would allow for the determination of demographic and environmental factors contributing to the injury, such as job type, tenure, training and experience, or lost time away from work. Second, there is a possibility that many patients injured in a rural location were missed due to being admitted to another hospital in the area. Also, it was possible that these rural patients sustained less severe injuries and were treated locally. Likewise, those workers killed at the site and not transported to the hospital were not represented in the analysis. Finally, the small sample size of the study population from a single institution limits the generalizability of the results.

## Conclusion

There is a growing need for enhanced surveillance of the onshore oil and gas extraction industry to understand risk factors for fatal and non-fatal injuries.^1^ To our knowledge, this is one of the first studies focusing solely on onshore oil rig injuries. Study results showed that extremity and head/facial injuries were the most common. In addition, most injuries were the result of patients being struck by an object or as the result of a fall. By targeting the main causes of injuries, training and prevention programs can be created to decrease the incidence of on-the-job injuries among this rapidly growing employment sector.

## Figures and Tables

**Table 1:** Patient demographics, injury severity, and injury details.

Variable	Percent (N)
Number of Patients	100.0% (66)
Age, years*	36.5 ± 11.8 (66)
Male	100% (66)
Race (Caucasian)	81.8% (54)
Injury Severity Score (ISS)*	9.4 ± 8.9 (66)
Initial Glasgow Coma Scale (GCS) Score*	13.8 ± 3.4 (66)
Abbreviated Injury Severity Score (AIS)*	
Head/neck	2.4 ± 1.1 (21)
Face	1.7 ± 0.6 (15)
Chest	2.6 ± 1.4 (11)
Abdomen	2.7 ± 0.8 (10)
Extremities	2.7 ± 0.7 (31)
External	1.1 ± 0.3 (40)
Type of Accident	
Struck	40.9% (27)
Fall	19.7% (13)
Caught in machine	12.1% (8)
Explosion	10.6% (7)
Pinned	9.1% (6)
Struck with subsequent fall	6.1% (4)
Cut	1.5% (1)

* Mean ± SD

**Table 2:** Injury characteristics.*

Injury Parameter	Percent (N)
Head Injury	
Loss of consciousness	16.7% (11)
Concussion	10.6% (7)
Skull fracture	6.1% (4)
Subarachnoid hemorrhage	4.5% (3)
Subdural hematoma	4.5% (3)
Facial Fracture	22.7% (15)
Spine Injury	
Lumbar	12.1% (8)
Thoracic	10.6% (7)
Cervical	1.5% (1)
Spinal cord injury	3.0% (2)
Thoracic Injuries	
Rib fracture	7.6% (5)
Pneumothorax	4.5% (3)
Hemothorax	1.5% (1)
Abdominal Injuries	
Urinary bladder	3.0% (2)
Spleen	1.5% (1)
Renal	1.5% (1)
Pelvic Fracture	7.6% (5)
Hip Fracture	1.5% (1)
Lower Extremity Fractures or Dislocations	25.8% (17)
Upper Extremity Fractures or Dislocations	18.2% (12)
Clavicle/Scapula	3.0% (2)
Burns	9.1% (6)

* A single patient could be subject to multiple injuries.

**Table 3:** Characterization of hospitalization details and disposition.

Hospital Parameter	Percent (N)
Number of Observations	100.0% (66)
Intensive Care Unit (ICU) Admission	34.8% (23)
ICU length of stay, days*	1.7 ± 2.5 (66)
Mechanical Ventilation	13.6% (9)
Mechanical ventilation days*	0.6 ± 2.3 (66)
Surgery	40.9% (27)
Permeant Disability	6.1% (4)
Hospital Length of Stay, days*	5.8 ± 16.6 (66)
Disposition	
Home	78.8% (52)
Rehabilitation	16.7% (11)
Nursing Facility	1.5% (1)
Death	3.0% (2)

* Mean ± SD
